# Clinician Maladaptive Anxious Avoidance in the Context of Implementation of Evidence-Based Interventions: A Commentary

**DOI:** 10.3389/frhs.2022.833214

**Published:** 2022-06-09

**Authors:** Emily M. Becker-Haimes, Corinna C. Klein, Hannah E. Frank, Maria A. Oquendo, Shari Jager-Hyman, Gregory K. Brown, Megan Brady, Miya L. Barnett

**Affiliations:** ^1^Department of Psychiatry, University of Pennsylvania Perelman School of Medicine, Philadelphia, PA, United States; ^2^Hall Mercer Community Mental Health, University of Pennsylvania Health System, Philadelphia, PA, United States; ^3^Department of Counseling, Clinical, and School Psychology, University of California, Santa Barbara, Santa Barbara, CA, United States; ^4^Department of Psychiatry and Human Behavior, The Warren Alpert Medical School of Brown University, Providence, RI, United States; ^5^Bradley Hospital, Lifespan Health System, Riverside, RI, United States

**Keywords:** clinician anxiety, mental health services, implementation, evidence-based intervention, exposure therapy, suicide prevention, time-out

## Abstract

This paper posits that a clinician's own anxious reaction to delivering specific evidence-based interventions (EBIs) should be better accounted for within implementation science frameworks. A key next step for implementation science is to delineate the causal processes most likely to influence successful implementation of evidence-based interventions (EBIs). This is critical for being able to develop tailored implementation strategies that specifically target mechanisms by which implementation succeeds or fails. First, we review the literature on specific EBIs that may act as negatively valenced stimuli for clinicians, leading to a process of clinician maladaptive anxious avoidance that can negatively impact EBI delivery. In the following sections, we argue that there are certain EBIs that can cause emotional distress or discomfort in a clinician, related to either: (1) a clinicians' fear of the real or predicted short-term distress the EBI can cause patients, or (2) fears that the clinician will inadvertently cause the patient harm and/or face liability. This distress experienced by the clinician can perpetuate a cycle of maladaptive anxious avoidance by the clinician, contributing to lack of or suboptimal EBI implementation. We illustrate how this cycle of maladaptive anxious avoidance can influence implementation by providing several examples from leading EBIs in the psychosocial literature. To conclude, we discuss how leveraging decades of treatment literature aimed at mitigating maladaptive anxious avoidance can inform the design of more tailored and effective implementation strategies for EBIs that are negatively valenced.

## Introduction

To be maximally effective, implementation strategy design should be guided by causal understanding of implementation processes and the determinants of clinician behavior {i.e., evidence-based intervention [EBI] use; (1)}. Refining current conceptual models of implementation to identify core targets for implementation strategy design is an important step for the field. In this paper, we propose that leading behavioral change theories in implementation science do not adequately account for the relationship between a clinician's own anxious distress and EBI implementation. Specifically, we propose that certain EBI techniques may act as negatively valenced stimuli for clinicians, leading to that EBI eliciting anxious reactions in clinicians; this may result in *clinician maladaptive anxious avoidance* of that EBI, which then interrupts the implementation process.

In this manuscript, we first define and review the rationale for focusing on clinician maladaptive anxious avoidance and articulate how it differs from other leading constructs in implementation frameworks such as attitudes and self-efficacy. We then draw from a robust literature examining processes of maladaptive anxious avoidance from the broader psychological field (2–6) to illustrate a conceptual model of how clinician maladaptive anxious avoidance can negatively impact EBI delivery. To demonstrate the potential applicability of these processes to mental health EBI implementation, we then provide several examples of how this process can unfold. We conclude by describing the implications of attending to maladaptive anxiety for the refinement of existing and generation of novel implementation strategies.

It is important to note that we are in no way arguing for the de-emphasis of attending to organizational and other contextual factors in the understanding of implementation processes. The importance of such factors is well-established [e.g., (7–9)]. However, individual clinician factors are known to interact with contextual variables to influence EBI implementation (10) and thus are critically important to attend to. We argue for the extension of current models of clinician factors in implementation to explicitly account for clinicians' emotional reactions associated with certain EBIs. Doing so will allow for implementation strategy development directly targeted at a promising, yet currently underemphasized, implementation determinant.

### Rationale for Focusing on Clinician Maladaptive Anxious Avoidance

**Figure 1:**
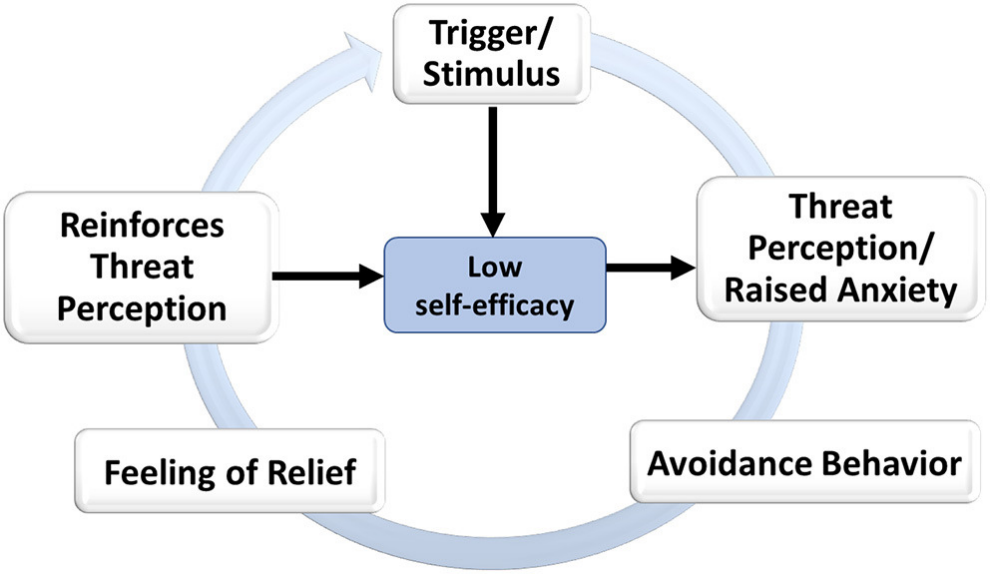
Traditional model of the cycle of maladaptive avoidance.

Maladaptive anxiety comprises unhelpful cognitions (e.g., threat overestimation) and avoidance behaviors related to feared stimuli. While the etiology of maladaptive anxiety is multifactorial (11–14), it is well-established that anxiety is maintained and worsened through continued avoidance of feared stimuli [“maladaptive anxious avoidance”; (15)]. This maladaptive anxious avoidance can be overt (e.g., situational avoidance or escape behaviors) or more subtle [e.g., cognitive avoidance or escape strategies, such as worry, rumination, or thought suppression; (2)]. Regardless of its presentation, maladaptive anxious avoidance contributes to or reinforces low self-efficacy to engage with these stimuli and, in turn, compounds anxiety [(16, 17); see Figure 1 for a traditional model of the cycle of maladaptive avoidance].

Although rarely directly referenced in leading implementation and behavioral change models, clinicians' own anxiety and distress about delivering a given EBI have been implicated in numerous studies. Specifically, clinician anxiety has been associated with suboptimal implementation in multiple studies of leading EBIs, most notably with three exemplar EBIs we discuss here: (1) exposure therapy for anxiety, trauma, and eating disorders, (2) the use of suicide screening, assessment, and brief interventions [e.g., the Safety Planning Intervention; (18)] for individuals at risk for suicide, and (3) the use of time out for pediatric disruptive behavior disorders.

We briefly review specific examples of how clinician anxiety has been highlighted as a driver of the research-practice gap for each of these three EBIs.

#### Exposure Therapy for Anxiety, Eating, and Post-traumatic Stress Disorders

Exposure therapy, which involves gradually supporting patients to face their fears and engage in behaviors they are avoiding, is arguably the most strongly empirically supported psychosocial EBI for anxiety and traumatic stress-related disorders (19–21) with strong emerging evidence for eating disorders (22, 23). However, it remains highly underutilized, with less than a third of clinicians routinely employing this EBI with patients likely to benefit (24, 25). There are multilevel barriers to exposure therapy implementation (26). However, the most robust predictors of exposure therapy's underutilization and suboptimal delivery include: (1) clinicians' negative beliefs, such as concerns about iatrogenic effects [e.g., (27, 28), and (2) clinicians' own *affective experiences*, such as anxiety about delivering the intervention (29–33). For example, clinicians who experience more anxiety during delivery of exposures are more likely to discontinue an exposure prematurely or coach the patient to engage in contraindicated arousal reduction strategies (e.g., deep breathing, progressive muscle relaxation) that undermine the intended goal of the exposure (30, 31). Importantly, two separate studies demonstrated that attempting to directly target clinician anxiety about exposures within the training context led to superior implementation outcomes compared to traditional training models (34, 35).

#### Suicide Screening, Assessment, and Brief Intervention for Suicide Risk

Clinician anxiety about working with patients at risk for suicide is also well-documented (36, 37). Patient death by suicide is the greatest fear clinicians report from a long list of possible adverse outcomes (38), and clinicians identify a range of anxieties about addressing suicide risk with patients (39–41) as barriers to effective implementation of suicide prevention EBIs (42). Critically, there is a paucity of research to suggest that training alone is sufficient to combat clinician anxiety. For example, a recent report in the state of Pennsylvania showed mandating training in suicide prevention for all licensed psychologists was not associated with a reduction of clinician-reported anxiety and distress related to working with suicidal patients. In fact, approximately a third of all survey respondents reported high levels of distress 4 years following the mandate, with fewer than 5 percent reporting minimal distress (43).

#### Time Out for Disruptive Behavior Disorders

Teaching caregivers to use time out is a core component of many behavioral parent training EBIs for young children (ages 2–8) with disruptive behavior disorders (44, 45), oppositionality, aggression, and attention deficit/hyperactivity disorder (46). When delivering an evidence-based time out, clinicians teach and coach caregivers to temporarily remove positive attention and reinforcement as a consequence of non-compliance or aggression. Time out has been shown to decrease problem behaviors for children and improve parenting when delivered appropriately in the context of a warm, reinforcing caregiver relationship (44, 47), and a meta-analysis of parenting interventions showed that time out is a treatment component that leads to larger effect sizes (45). However, time out is underutilized within clinical practice. A recent study of the implementation of multiple EBIs in community settings found that clinicians used time out less than almost all other typical components of behavioral parent training [e.g., praise, tangible rewards, logical consequences; (48)]. Less empirical attention has focused on time out relative to exposure therapy and suicide prevention EBIs with respect to clinician anxiety. However, clinician aversion to implementing time out in sessions is a known barrier to its implementation. For example, in a study of Parent-Child Interaction Therapy (which includes time out as a core intervention technique), clinicians reported their own negative beliefs and described time out as unacceptable to their colleagues, both of which hindered their implementation of the parenting protocol. In qualitative reports, clinicians reported worrying that time out might worsen child anxiety and behavior (49). Although this fear has been disputed in the literature (44, 50), there is evidence it leads to clinician avoidance of the procedure altogether.

It is important to note that not all anxious avoidance is maladaptive (51). Clinically, *maladaptive* anxious avoidance is best conceptualized as an avoidance behavior that contributes to the maintenance of functionally impairing anxiety symptoms [i.e., the function of the behavior is maladaptive or illogical; (51)]. This does not apply to avoidance behaviors that are fundamental to keeping someone safe, healthy, or emotionally well. For example, fear activation in the presence of a clear and dangerous threat (e.g., a poisonous spider, nearby community violence), should lead to avoidance to maintain individuals' safety. Within the implementation context, we can conceptualize maladaptive anxious avoidance as occurring when clinical decision making is primarily driven by a clinician's own affective reaction to a given EBI, rather than by the best interests of the patient, leading to avoidance of delivering some or all of that EBI. In contrast, adaptive anxious avoidance may arise when a clinician experiences discomfort at the thought of delivering a given EBI in a session where a patient has recently learned of the death of a close family member; this is likely an appropriate emotional reaction that should drive clinician decision-making to adjust the treatment plan (52).

To date, research characterizing clinician anxiety has been isolated to individual EBIs, rather than conceptualizing clinician anxiety as a broader potential implementation determinant. Our collective experiences training and supporting clinicians to deliver these three EBIs referenced above are also consistent with the literature. We all have observed clinicians closing their eyes or expressing discomfort upon viewing video examples of certain EBIs in action. Further, we have encountered clinician discomfort around implementing these EBIs within the context of ongoing consultation. Consider this illustrative [paraphrased] quote from a clinician who attended a 12-h workshop that combined didactic and experiential training about exposure therapy for anxiety disorders: “*You've convinced me that this is a great treatment approach for anxiety. I can see how it would work. But I'm never going to do it, I just don't think I can, it would stress me out too much.”* Delineating the causal processes that lead to clinician maladaptive anxious avoidance and how it influences implementation will inform future efforts to implement negatively valenced EBIs. Anecdotally, many of us also have begun attending to clinician emotional anxiety and distress within trainings with success, underscoring the importance of optimizing strategies that attend to clinician anxiety directly. Organizing future training efforts within a theoretically-guided framework is likely to facilitate implementation success.

### Differentiating Maladaptive Anxious Avoidance From Leading Constructs in Implementation Frameworks

There are several closely related constructs reflected in implementation frameworks {e.g., the Consolidated Framework for Implementation Research [CFIR] (7)}. At the individual level, these include clinician attitudes or beliefs about a specific intervention and clinician self-efficacy regarding delivering a given EBI. Attitudes relate to how positively one feels about using a specific EBI or EBIs in general (53). For example, the Evidence-Based Practice Attitude Scale (EBPAS) was developed to measure how community clinicians perceive using “new types of therapy/interventions,” that are manualized or developed by researchers. The EBPAS includes domains related to how appealing the intervention is, if there are requirements to deliver the intervention (e.g., agency mandates), the clinician's openness to change, and the perceived divergence between the new practice and the clinician's usual practices (54). Other measures of clinician attitudes compare the advantages of using different interventions, as multiple studies have demonstrated that attitudes vary based on the characteristics of EBIs (55–57).

Despite at times incorrectly being used interchangeably (53), attitudes and self-efficacy are distinct constructs. While attitudes relate to beliefs about the EBI, self-efficacy is defined as an individual's beliefs about their own skills and capabilities to administer an EBI (58). Perceived self-efficacy with an EBI may relate to a clinician's willingness to deliver an EBI and their fidelity to the model (59). Both attitudes and self-efficacy are closely related to, yet distinct from, clinician maladaptive anxious avoidance. Bandura's (58) social cognitive theory posits that when individuals perceive themselves as ineffective, they are more likely to have fearful expectations or engage in avoidance, which in turn can lower self-efficacy. Additionally, attitudes or beliefs that a treatment is harmful may reinforce avoidance. However, current measurement of EBI attitudes and self-efficacy and conceptual models of implementation processes rarely account for the clinician's own experiences of anxiety around EBI delivery.

At the intervention level, attention has been paid to the impact of an intervention's salience and complexity on implementation (7, 60). Emerging work also suggests that characteristics of specific EBIs likely influence a clinician's motivation to deliver it (61, 62). However, to our knowledge, no implementation frameworks include *EBI valence* (or the emotional reaction elicited by an EBI for a clinician) as an intervention characteristic of interest.

### Applying the Cycle of Maladaptive Anxious Avoidance to Clinician EBI Implementation

**Figure 2:**
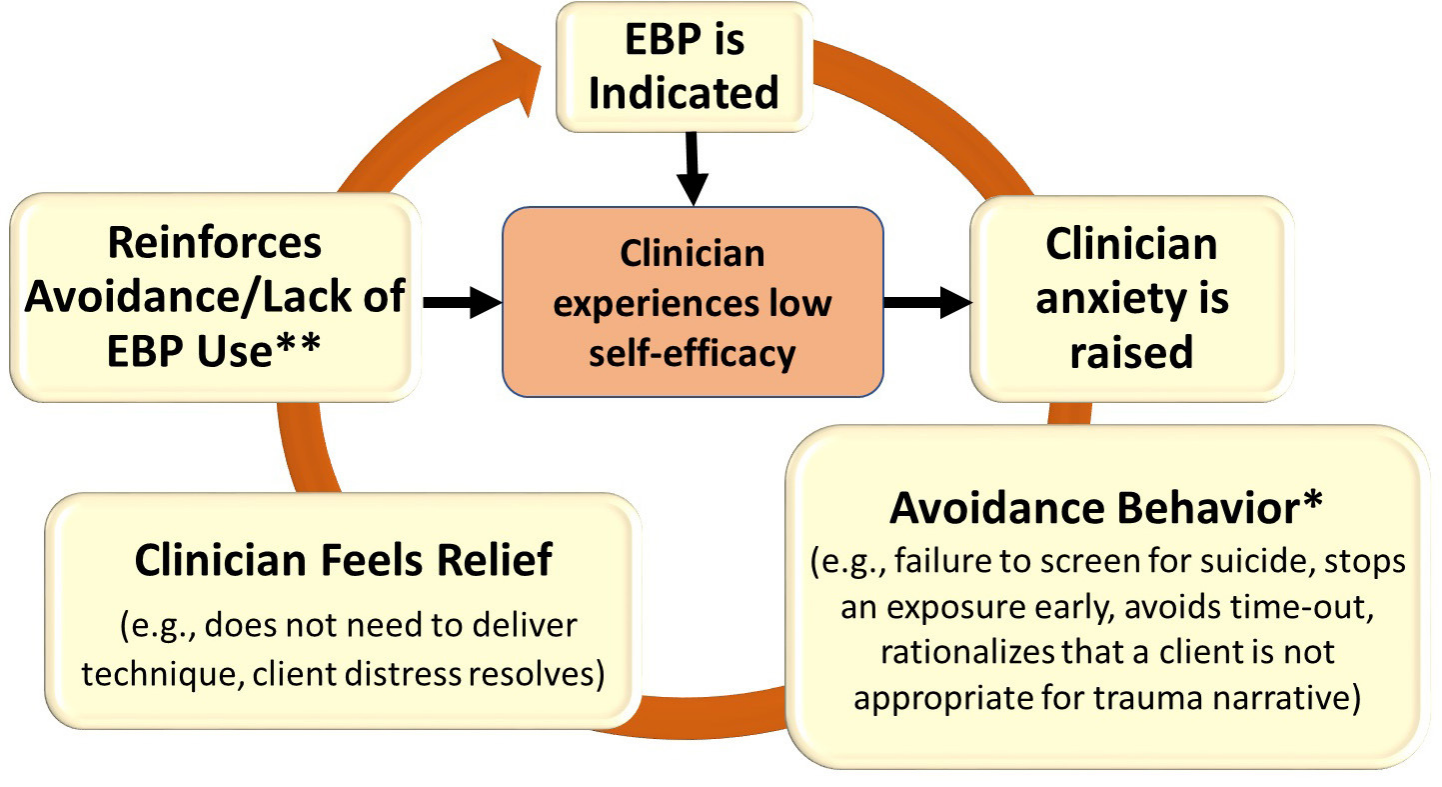
Application of the conceptual cycle of avoidance model to maladaptive clinician anxiety related to EBI use. ^*^We propose that initial avoidance behaviors can occur at the point of *intention formation* or during the delivery of the EBP itself; in this latter case, we propose that clinician avoidance may reduce clinician intentions to subsequently use the EBP. ^**^In some cases, this may also be clinically iatrogenic. For instance, if a patient stops an exposure early due to a patient's distress in the moment, it can reinforce a patient's perception that they are unable to tolerate their distress or handle the given situation. Similarly, if a clinician ends a time-out early due to a patient's extreme dysregulation, it may reinforce the patient's initial behavior that prompted a time-out sequence. Clinicians are also unable to experience reduction in their *own* distress if the intervention technique is followed through successfully to completion.

Figure 2 illustrates how the cycle of maladaptive anxious avoidance can be applied to clinician EBI delivery. The cycle described begins when a negatively valenced EBI is clinically indicated (e.g., a clinician is treating an anxious patient, routine suicide screening is rolled out in an organization, a caregiver cannot manage their young child's aggression) and leads to some sort of anxious distress response within the clinician. We define an EBI of negative valence as: *an EBI that can cause emotional distress or discomfort in a clinician, related to: (1) the real or predicted short-term distress the EBI can cause patients, or (2) fears that the clinician will inadvertently cause the patient harm or face liability. The clinician's emotional distress in turn is associated with negative beliefs about the intervention being harmful or inappropriate*. All three of the psychosocial EBIs mentioned above (exposure therapy, time out, and suicide prevention screening, assessment, and intervention) meet this definition of a negatively valenced EBI.

Importantly, not all EBIs are negatively valenced. Consider, for example, relaxation or grounding techniques. Such techniques are easy to learn and use and are relatively low-risk. They can be conceptualized as positively valenced EBIs, as they often have the immediate effect of reducing patient distress, which in turn is rewarding and satisfying for clinicians. Consistent with this, studies examining practices among clinicians who were trained through CBT implementation efforts suggest that relaxation is often a treatment practice that is quickly incorporated by clinicians and the practice is sustained over time (24, 63, 64). This suggests that clinicians may be more likely to implement positively valenced EBIs.

A clinician's anxious reaction to a negatively valenced EBI may occur during the point of intention formation (e.g., during training) or when the clinician is considering or attempting to deliver the EBI. When this anxiety is also accompanied by low self-efficacy or confidence in the ability to handle or cope with the anxiety-provoking situation, we propose this subsequently leads to an urge for the clinician to engage in some type of avoidance behavior to obtain emotional relief. EBI avoidance behaviors may take several forms. For example, a clinician may simply hold low intentions and never implement the EBI, thus avoiding ever experiencing any associated distress (an overt and immediate avoidance). Alternatively, the clinician may initially have high intentions to deliver an EBI; however, when they begin to deliver the EBI they find it so aversive that they discontinue its implementation (an overt and delayed avoidance) to obtain relief. Avoidance may also take subtler forms, such as misidentifying a patient as not appropriate for a specific EBI (65) or rationalizing that a patient with whom it may be more challenging to deliver a specific EBI may not be appropriate for that intervention (66). Critically, removing negatively valenced components from the broader evidence-based protocols from which they were derived (e.g., time out from behavioral training programs) and only delivering positively valenced components of EBIs, such as teaching parents to praise their child, can compromise fidelity and clinical outcomes (67).

Regardless of a clinician's initial intentions to use the EBI, avoidance may perpetuate a cycle of further maladaptive anxious avoidance. Relief associated with avoidant coping can serve as a powerful negative reinforcer of both negative beliefs about the EBI (e.g., “This intervention doesn't work”) or the clinicians' own self efficacy (e.g., “I am not skilled enough to deliver this intervention”). As such, clinicians' self-efficacy and their intentions to deliver the EBI either remain low or are further lowered over time, leading to lack of or suboptimal EBI implementation. To illustrate this process, we provide examples from the three leading EBIs described above. We explicate how clinician anxiety can negatively interfere with the implementation process for each EBI and how this may be clinically iatrogenic in some instances.

#### Exposure Therapy

Exposure processes involve intentionally provoking distress in patients to help them learn that a feared outcome may not come true or may be more tolerable than predicted. Critically, the distress patients experience during exposures may or may not decrease in a given therapy session for the practice to be considered successful (68, 69). This means clinicians delivering exposure must often tolerate the fact that their patient may be dysregulated and highly anxious for a short period of time during the exposure practice. As noted above, when clinicians experience distress about this, they are more likely to deliver exposure suboptimally; most commonly, this takes the form of a clinician reducing the intensity of the exposure to make it easier for a patient or abandoning the exposure altogether (i.e., avoidance). When the exposure intensity is reduced in this way, the patient and the clinician may both experience immediate relief— the patient is no longer engaging in the stressful task and the clinician no longer needs to tolerate their patient's distress. This is concerning for two reasons. First, it can be clinically iatrogenic by reinforcing a patient's perception that they are unable to tolerate their distress or handle the given situation [i.e., reduces intervention efficacy; (70)]. Second, the clinician also does not get the opportunity to see the full exposure effect occur (i.e., the patient ultimately is able to tolerate the distress and experience long-term symptom relief); this perpetuates the cycle of clinician avoidance. It is possible that this may contribute to why exposure has been identified as one of the most difficult EBIs to sustain in community settings over time (64).

#### Suicide Screening, Assessment, and Brief Intervention

Clinicians may report experiencing anticipatory anxiety about screening patients for suicide risk [e.g., fear of patients endorsing suicidal risk and not knowing how to intervene, not having enough time to intervene, or concern that asking about suicide will increase risk; (39–41). In such cases, clinicians may avoid asking about suicide altogether or ask in inappropriate ways that limit the likelihood of identifying a patient at risk for suicide [e.g., “you're not thinking about suicide, right?”; (40)]. This provides the clinician with short-term relief associated with not “discovering” suicide risk, although it may ultimately place the patient at higher risk for suicide. When clinicians do engage in suicide prevention EBIs, they also may worry about underestimating a patient's risk level (e.g., incorrectly deeming a patient safe to go home), fearing that the patient will attempt suicide or that they will be liable for making the wrong decision. This may lead to avoidance behaviors such as implementing ineffective practices [e.g., “no suicide contracts;” (71)] or over-referring patients to emergency rooms or crisis centers, which in turn may lead to hospitalization. In response, the clinician likely experiences short-term relief knowing that their patient is safe in an inpatient facility, rather than needing to tolerate the discomfort and uncertainty of sending a high-risk patient home with a safety plan. However, this is problematic for at least two reasons. First, there is limited evidence that no-suicide contracts or inpatient hospitalization reduces suicide risk [and they may even have iatrogenic effects; (71–73)]. Second, the clinician loses out on the opportunity to learn that they can safely send a high-risk patient home with an adequate safety plan in place, thus perpetuating the likelihood that they will continue to experience anxiety about working with individuals at risk for suicide in the future.

#### Time Out

Clinician concerns that time out may cause harm to youth (47, 49) may contribute to clinicians avoiding the procedure altogether or presenting the technique to families as something unlikely to be helpful to their child (74). Some clinicians who attempt to deliver the EBI may experience negative reactions and emotional distress in response to the temporary increased distress exhibited by children (and often caregivers) when first implementing time out. Specifically, child emotion dysregulation (e.g., crying, screaming) can intensify during timeout when it is first used. This immediate increase in emotion dysregulation and a temporary extinction burst in disruptive behaviors during the initial use of time out in children can be distressing for caregivers and clinicians. Although research to date has not investigated clinician anticipatory anxiety about eliciting caregiver and child distress through use of time out, it likely contributes to clinician reluctance to implement it in session, thereby depriving many families of an effective element of treatment for disruptive behavioral disorders in young children.

In addition to avoiding time out altogether, clinician anxiety during a time out sequence may lead to abandoning the procedure midway, further reinforcing the child's disruptive behaviors and dysregulation. When first implementing the time out procedure with a young child, the clinician must tolerate both the child's and caregiver's distress. The intensity of a child's dysregulation and the behaviors that often accompany it may cause an anxiety-driven response in the clinician to abandon the time out sequence to alleviate their own, the caregiver's, and the child's distress. This provides immediate relief, but ultimately reinforces the behaviors time out intends to decrease (aggression and defiance) and denies the child an opportunity to self-regulate. To be effective, the end of time out must be contingent on the child displaying appropriate behaviors and emotion regulation (44). A premature interruption of the sequence due to clinician anxiety can detrimentally strengthen child disruptive behavioral symptoms (i.e., worsen symptoms).

#### Beyond Psychosocial EBIs

Of note, we have so far only discussed implementation processes for psychosocial EBIs. However, these processes likely occur in the broader healthcare space as well. For example, physicians are known to experience discomfort engaging in “goals of care” conversations around end of life (e.g., advanced directives), which may lead to their avoidance of such conversations (75). Further delineating what other EBIs also constitute negatively valenced EBIs is an important next step for this line of research.

### Implications for Implementation Strategy Design

Taken together, there is compelling evidence that clinicians' maladaptive anxious avoidance processes can impede implementation of specific EBIs. This suggests the need for strategies that target clinician anxiety associated with EBIs to optimize implementation and effectiveness. To accomplish this goal, we can draw on decades of research on behavior change for patients with anxiety to target *clinician* anxiety about EBI delivery. Specifically, we propose that implementation strategies that leverage principles of exposure therapy to reduce clinician maladaptive anxious avoidance may be particularly promising. As described above, exposure therapy is a well-established technique for teaching individuals to cope with and reduce maladaptive anxious avoidance. Although exposure is a negatively-valenced EBI, it is well-tolerated by patients, has few side effects (76), and a robust literature supports the utility of brief, exposure-based treatments to target specific fears [in this case, fears about delivering EBIs; (77, 78)].

We propose that each of the four standard phases of exposure therapy can be incorporated into an implementation strategy to target clinician anxiety: psychoeducation, assessment/hierarchy building, guided practice, and relapse prevention. For example, an implementation strategy that leverages exposure principles might include: explicitly labeling an expectation that a given EBI may elicit anxious distress in a clinician that may lead them to want to avoid its delivery (psychoeducation); assessing an individual clinician's specific fear(s) related to its delivery and identifying which fears are most intense and likely to interfere with implementation (assessment/hierarchy building); engaging in targeted practice—either imaginal or *in vivo*—to directly address the clinician's specific feared outcomes about use of the EBI (guided practice); and setting intentions and discussing how to plan for and manage expected distress related to implementation (relapse prevention). During the guided practice sessions, an exposure frame purports the potential utility of trainers (1) identifying core fears and anticipated anxiety (e.g., via subjective units of distress [SUDS]), (2) engaging in targeted practice to violate assumptions of core fears and track SUDS changes, and (3) engaging in targeted cognitive debriefing to enhance coping self-efficacy regarding a clinicians' ability to deliver the EBI.

Indeed, recent data suggest that an exposure-based strategy comprising the above elements to address clinician anxiety may improve implementation outcomes. Specifically, Frank et al. (35) conducted a pilot feasibility trial in which clinicians were randomized to a novel training paradigm (didactics + exposure experience) or traditional training [didactics + role-play practice, a gold-standard component of EBI training and consultation; (79)]. In the novel paradigm, clinicians both delivered *and received* exposure therapy, guided by a one-session treatment model (77, 80). A key difference between the exposure-based approach in the novel condition and the use of role plays in the traditional training arm was that the exposure-based condition comprised a series of structured steps designed to facilitate individual identification of primary fears, practice that gradually increased in intensity, and cognitive processing after each practice attempt. In contrast, role plays were specifically focused on allowing the clinician to practice the skill in a simulated environment and receive feedback on their technique (35). While this novel paradigm was initially conceptualized primarily to enhance experiential learning (rather than specifically targeting clinician anxiety), mixed-methods findings suggested that the use of exposure directly addressed clinician anxiety and bolstered self-efficacy about EBI implementation. Notably, clinicians who entered the study with a higher baseline level of personal anxiety reported being most likely to benefit from the exposure practice, citing that it helped them learn what it was like to be a patient receiving the treatment {i.e., that it was tolerable and helpful; interestingly, this is very much in line with how patients often describe exposure [e.g., (81)], despite clinicians' beliefs that exposure may be perceived by patients as damaging or traumatic}. Clinicians in the novel paradigm also demonstrated higher exposure use at 1-month follow-up compared to the traditional training group (35).

Exposure-based implementation strategies have not yet been conceptualized as a broader framework that can guide implementation strategy design. However, the success of Frank et al. (35) and the robust literature reviewed above suggests exciting areas for future research in implementation strategy design that leverage exposure therapy principles to improve implementation of negatively valenced EBIs. There are key open questions worth highlighting. First, it will be important to elucidate at what point in a clinician's developmental trajectory an exposure-based implementation strategy would ideally be deployed to be most effective. For example, is an exposure-based implementation strategy to support clinician use of a negatively valenced EBI equally as effective if employed as a part of ongoing continuing education for licensed clinicians as it is when employed in the early graduate stage of training when a clinician is first introduced to the EBI? Second, can a universal approach, whereby all those learning to deliver a negatively valenced EBI do so within the context of an exposure-based frame, be successful? Or does an exposure-based implementation strategy need to be targeted specifically to those clinicians who express anxiety or distress at the thought of delivering that EBI to be successful?

Third, future trials testing the impact of an exposure-based implementation strategies must determine if such efforts can be effective when targeted to individual clinicians or would ideally occur within the context of organizational efforts to roll out negatively valenced EBIs to all eligible patients. Advancing understanding of how and when to deploy an exposure-based implementation strategy must occur within the context of the broader implementation landscape in mental health. As noted above, an exposure-based implementation strategy is not intended to address the contextual and systems-level barriers that also impede successful implementation efforts. Integrating future design and evaluation of exposure-based implementation strategies for negatively valenced EBIs within broader implementation frameworks that more fully account for these contextual implementation determinants {e.g., the Exploration, Preparation, Implementation, Sustainment Model [EPIS; (82)] or the Consolidated Framework for Implementation Research [CFIR; (7)]} is recommended to fully advance the reach of negatively valenced EBIs.

Finally, there are important challenges related to evaluating the effects of an exposure-based implementation strategy and its eventual scale-up worth noting. Evaluating the success of mental health implementation success has been historically challenged by the private, in-person therapy environment, which provides limited opportunities for oversight and monitoring of whether EBIs are delivered. The rapid expansion of telehealth in response to the COVID-19 pandemic [e.g., (83)] as well as innovations in technology to support fidelity monitoring of EBI delivery [e.g., (84)], provide exciting opportunities to overcome these challenges. Future design and deployment of an exposure-based implementation strategy would ideally be explored in tandem with technological innovations to: (1) optimally assess impact, and (2) inform future refinements to enhance its efficacy at improving clinician uptake of negatively valenced EBIs. To promote eventual scale-up to the many practicing clinicians who might ultimately benefit from an exposure-based model of training, future work might consider partnering with virtual reality experts to translate exposure-based scenarios for negatively valenced EBIs to a readily deployable virtual reality platform. This would reduce the person-power needed to administer the exposure-based components of an implementation strategy and could ultimately be used widely as a standard adjunctive component to training and consultation.

## Conclusions

Designing and testing scalable implementation strategies to increase EBI use is critical for ensuring that those experiencing psychiatric distress receive effective treatment. Extending current conceptual models of implementation to also consider the role of clinician maladaptive anxious avoidance specifically has promise for improving the design of training content for EBIs prone to provoking distress in clinicians. In this conceptual paper, we have argued that there are core EBIs that hold high *negative valence* for clinicians and contribute to maladaptive anxious avoidance that interferes with effective implementation. Interestingly, the EBIs used as exemplars in this paper (exposure therapy, suicide prevention techniques, and time out) are some of the EBIs with the strongest empirical support for their efficacy, and yet are rarely used. Simply put, one could argue that the EBIs with the widest research-practice gaps are those that are negatively valenced, underscoring the importance of understanding how an EBIs' valence influences implementation. Attending directly to clinicians' maladaptive anxious avoidance in the context of implementing negatively valenced EBIs holds exciting potential to overcome barriers to leading EBIs that remain sorely underutilized in clinical practice.

## Data Availability Statement

The original contributions presented in the study are included in the article/supplementary material, further inquiries can be directed to the corresponding author/s.

## Author Contributions

EB-H, MBa, and CK drafted portions of the manuscript. HF, MO, SJ-H, GB, and MBr critically reviewed and edited the manuscript. All authors contributed substantially to conceptualization of constructs delineated in this manuscript and reviewed and approved the final edited draft for submission.

## Funding

This work was supported by P50 MH127511 (MPIs: Brown, Oquendo). HF's work was supported by T32MH019927.

## Conflict of Interest

MO and GB receive royalties from the Research Foundation for Mental Hygiene for the commercial use of the Columbia Suicide Severity Rating Scale. MO serves as an advisor to Alkermes, Otsuka, Mind Medicine, Sage Therapeutics, St. George's University and Fundacion Jimenez Diaz and her family owns stock in Bristol Myers Squibb. GB serves as an advisor for Oui Therapeutics, LLC and Psych Hub, LLC. The remaining authors declare that the research was conducted in the absence of any commercial or financial relationships that could be construed as a potential conflict of interest.

## Publisher's Note

All claims expressed in this article are solely those of the authors and do not necessarily represent those of their affiliated organizations, or those of the publisher, the editors and the reviewers. Any product that may be evaluated in this article, or claim that may be made by its manufacturer, is not guaranteed or endorsed by the publisher.
